# Reactive versus Constitutive: Reconcile the Controversial Results about the Prognostic Value of PD-L1 Expression in cancer

**DOI:** 10.7150/ijbs.33297

**Published:** 2019-07-21

**Authors:** Dapeng Hao, Guangyu Wang, Weiwei Yang, Jinan Gong, Xingmin Li, Mingming Xiao, Lijie He, Li Wang, Xiaobo Li, Lijun Di

**Affiliations:** 1 Department of Pathology, Harbin Medical University, Harbin, China.; 2 Faculty of Health Sciences, University of Macau, Macau, China; 3 Department of Gastrointestinal Medical Oncology, the Affiliated Tumor Hospital of Harbin Medical University, Harbin, China.; 4 Shuwen Biotech Co. Ltd., Deqing, China.; 5 Department of Pathology, People's Hospital of Liaoning Province. Shenyang, China.; 6 Department of Medical Oncology, People's Hospital of Liaoning Province. Shenyang, China.

**Keywords:** PD-L1, CTL, cancer, immunotherapy, microenvironment

## Abstract

The prognostic value of programmed death-ligand 1 (PD-L1) has been controversial in recent studies. PD-L1 is known to play a major role in suppressing the immune response, yet increasing studies have reported that PD-L1 expression has a favorable prognostic value for cancer patients. This raises the concern about how to understand PD-L1 expression: merely an immune inhibitory signal, or more likely a reactive process to T-cell response that indicates cytotoxic T lymphocyte (CTL) level in a tumor? To solve this dilemma, an integrative investigation is required. We compared the PD-L1 expression between tumor cells and immune cells, and characterized the inter- and intra-tumor correlation between CTL and PD-L1 expression. The prognostic values between PD-L1 and CTL is compared across 15 solid cancers and 11 independent cohorts of ovarian cancer. PD-L1 and PD-L1-adjusted CTL are analyzed in immunotherapy dataset receiving nivolumab. We observed unexpected high concordance between the prognostic value of PD-L1 and CTL across different cancers and cohorts. We found primarily reactive rather than constitutive PD-L1 expression in most tumors. We revealed that PD-L1-adjusted CTL level, rather than the expression of PD-L1, effectively predicts the responders to immune checkpoint inhibitors. This study highlights the importance of PD-L1 expression, as primarily a signature of reacting efficiency of pre-existing anti-tumor immunity, in balancing the tumor microenvironment. Importantly, it suggests that the reactive efficiency of PD-L1 is more useful to predict the response to immunotherapy.

## Introduction

Recent studies regarding the prognostic value of programmed death-ligand 1 (PD-L1) have put us into a dilemma. On one hand, PD-L1 elicits immune inhibitory signals, causes T-cell exhaustion and is expected to be associated with poor outcome 1. On the other hand, PD-L1 expression can be induced by interferon gamma (IFN-γ) and thus indicate the CTL in tumors and should be a favorable prognostic factor in cancer 2. The solution to this dilemma depends on how PD-L1 expresses in a tumor. Expression of PD-L1 can either be a reactive process of T cell response 3,4, in which case it represents the pre-existing anti-tumor immunity, or be regulated by cancer cell-intrinsic mechanisms and represents tumor-intrinsic immune resistance. Therefore, the relative importance of reactive vs. constitutive PD-L1 expression on prognosis needs to be investigated.

Among many inhibitory mechanisms of anti-tumor immunity, the PD-1/PD-L1 axis has drawn intensive efforts to understanding its prognostic significance and its therapeutic potential. In earlier studies, PD-L1 expression is often reported to be associated with poor prognosis of cancer patients 1. In recent years, however, more and more studies reported the favorable prognostic value of PD-L1 expression in cancer patients. For instance, in ovarian cancer, some researchers found that PD-L1 expression is primarily determined by tumor-infiltrating leucocytes (TILs) and associated with favorable outcome 5, whereas a previous study reported that PD-L1 is constitutively expressed by tumor cells and associated with poor prognosis 6. In non-small cell lung cancer (NSCLC) and in breast cancer, both favorable prognosis 7-9 and poor prognosis 10,11 were reported to be associated with PD-L1. In colorectal cancer (CRC), high expression of PD-L1 was found in microsatellite instable (MSI) tumors, which have high TILs and relatively favorable prognosis 12.

In this study, we characterized the inter- and intra-tumor association of CTL level and PD-L1 expression using pan-cancer samples. We demonstrated that the vast majority of tumor samples, even the tumors with PD-L1 amplification, show an inducible rather than a constitutive PD-L1 expression. The prognostic values of PD-L1 and CD8A are highly correlated and interactive across cancer types. Given this fact, the appropriate way to evaluate the prognostic value of PD-L1 is how efficiently it reacts to CTL rather than its expression level. Thus, PD-L1-adjusted CTL level in a tumor is especially vital for the PD-1/PD-L1 blockade immunotherapy.

## Materials and Methods

### Human samples

A total of 61 formalin-fixed paraffin-embedded cancer tissue samples, including 41 colorectal cancers, 16 breast cancers, 3 lung cancers and 1 esophageal cancer, were obtained from the Department of Pathology at the Third Affiliated Hospital of Harbin Medical University between 2009 and 2017. The written informed consent was obtained from all patients. This study was approved by the Ethics Committee of Harbin Medical University. The clinical information of these patients is shown in supplementary table 1.

### Immunohistochemistry (IHC) staining

5 µm-thick serial sections of cancer tissues were prepared, and the antigen retrieval was performed by heating in the citrate buffer (pH 6.0). To detect the PD-L1 expression and CTL-infiltrating in tumor tissues, the sections were incubated with a primary antibody against CD8 (ZSGB-BIO, China) or PD-L1 (Shuwen Biotech, China; it has been validated with Dako 22C3, Supplementary Fig.1) overnight at 4 ℃, and then were incubated with the HRP-conjugated goat anti-rabbit secondary antibody at room temperature for 30 min. After staining with DAB (ZSGB-BIO, China) and counterstaining with hematoxylin, the slides were recorded using Leica Microsystems Wetzlar (Leica-DM6600B). The area containing the expression of CD8A or PD-L1 in each tumor tissues was plotted by two pathologists independently, whereas the positive rate of CD8 positive cells or PD-L1 positive cells in each sample was automatically calculated by ImageScope software (Aperio, USA).

### PD-L1 expression in cancer cell lines and immune cells

Transcriptome data of immune cells were downloaded from GEO database (GSE22886) 13. Only the microarray data of Affymetrix human genome U133B platform in this dataset were analyzed because this array platform detects the expression of PD-L1. Transcriptome data of cancer cells were downloaded from Cancer Cell Line Encyclopedia (CCLE) database 14. Raw data were processed using the RMA method and quantile normalized. The same probe set of Affymetrix array (227458_at) was used for indicating the expression of PD-L1.

### Pan-cancer data of TCGA

Public available data of TCGA, including clinical information and copy number variation (CNV) data were downloaded from GDAC (http://gdac.broadinstitute.org). Copy number amplification or deletion of PD-L1 was determined by GISTIC 2.0 software and documented as “2” and “-2”, respectively. RNAseq-derived gene expression data of 9,264 TCGA tumor samples were downloaded from GEO (GSE62944) 15. This dataset provided the gene expression data that were reprocessed using the same pipeline for the comparison between tumor samples. We used the log2 transformed fragment per kilobase per million (FPKM) as the gene expression value. Samples from different portions of the same tumor were determined by the sample barcode of TCGA tumor samples and are provided in Supplementary Table 2. Gene expression from repeated samples of the same portion was averaged before analysis.

### Independent ovarian cancer datasets

Transcriptome datasets of serous ovarian cancer were obtained by database searches through PubMed, Array Express and GEO. We required that all the datasets detected the expression of PD-L1 and had at least 40 primary tumor samples of serous ovarian cancer with overall survival information. We got 11 datasets consisting of totally 1,312 patients. The processing of the microarray datasets has been described in our previous study 16. The RNAseq dataset OV.AU containing 80 primary serous ovarian tumors was obtained from a recent study 17.

### Statistical analysis

Standard statistical tests including Student T test, Pairwise T test, Wilcoxon rank sum test, Fisher exact test, Cox proportional hazard regression were used for the analysis of clinical data and genomics data, and a two-sided P< 0.05 was considered significant. Hazard ratio (HR) of continuous expression value was used to estimate the prognostic value of PD-L1 and CD8A. Meta-analysis was performed using R package “metafor”. All the analyses were performed in R 3.3.1.

## Results

### The concordance of prognostic values between PD-L1 and CD8A

**Figure 1:**
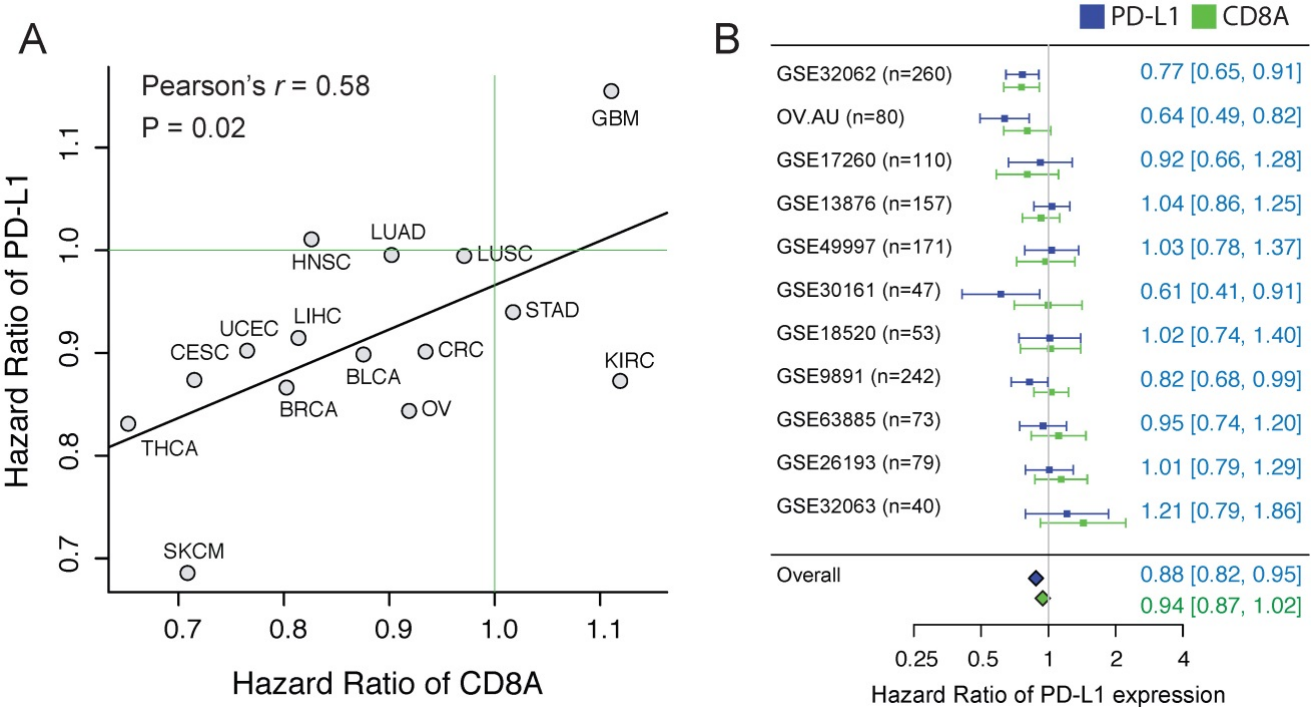
**The correlation of the prognostic values between CD8A and PD-L1.** (A) Scatter plot of hazard ratios of PD-L1 and CD8A across different cancers. (B) Forest plot visualizing the hazard ratios of univariate Cox proportional regression analyses of CD8A and PD-L1 expression in 11 independent ovarian cancer cohorts. The diamonds shows the fixed-effects meta-analysis summary of hazard ratios over 11 cohorts.

First, to investigate whether CTL and PD-L1 show different associations with clinical outcome, we estimated the CTL level using the expression level of CD8A, and evaluated the prognostic value of CD8A and PD-L1 expression in 15 solid TCGA cancer types using Cox proportional hazard analysis (Cox-PH). Interestingly, we observed a favorable prognostic value (hazard ratio (HR) < 1) in the majority of cancer types for both PD-L1 and CD8A (Supplementary Figure 2). Importantly, HR of the two genes seems to be highly correlated across different cancer types (Figure 1A. Pearson's r = 0.58, p = 0.02). In some cancers, PD-L1 is even more significantly associated with favorable outcome than CD8A. For example, in ovarian cancer, the HR of PD-L1 is 0.84 [95%CI: 0.74-0.96], whereas the HR of CD8A is 0.92 [95%CI: 0.81-1.04].

To further confirm using independent datasets, we collected to our knowledge all the published ovarian cancer datasets (n>=40) that have detected the expression of PD-L1 and CD8A. Considering the histological heterogeneity of ovarian cancer, only the tumors of serous type were included, consisting of totally 1,412 samples. Although the prognostic value of PD-L1 expression shows a high variation across cohorts, we observed a high concordance of the HRs between the PD-L1 and CD8A (Pearson's r = 0.64, p < 0.03). We applied a meta-analytic strategy to leverage different datasets, and found that PD-L1 expression is significantly associated with favorable outcome in ovarian cancer (Figure 1B, HR = 0.88 [CI%: 0.82-0.95], p = 0.02).

### Intrinsic PD-L1 expression in tumors

**Figure 2:**
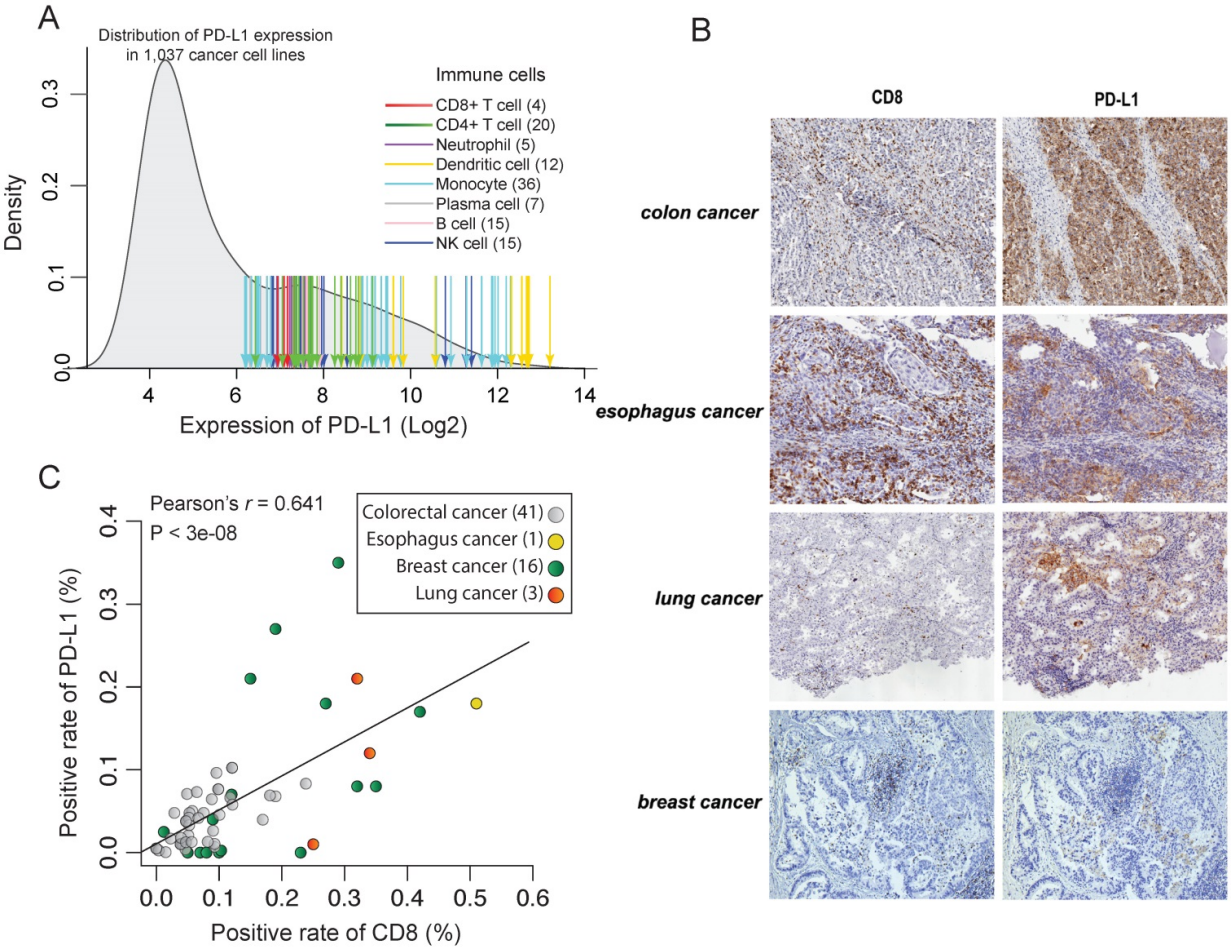
** Inter-tumor association of the expression between PD-L1 and CD8A.** (A) Density distribution of PD-L1 expression in 1,037 cancer cell lines and the values of PD-L1 expression in immune cells. Arrows indicate the expression value of individual immune cells, with the color corresponding to different immune cell types. (B) IHC staining of representative tumor tissue samples. (C) Correlation of positive rate between CD8+ cells and PD-L1+ cells across tumor samples, according to image analysis with pathologist scoring.

To test intrinsic PD-L1 expression in tumor cells, we first compared the expression level of PD-L1 between cancer cell lines and the immune cells that show a constitutive expression of PD-L1. To be consistent, the transcriptome data of immune cells and cancer cell lines were processed using the same method, and we used the same microarray probe ID to indicate the expression of PD-L1. Compared to the average expression of PD-L1 in immune cells, the intrinsic PD-L1 expression in cancer cell lines is on average 2^3.83^=14.2 times lower (Figure 2A). Notably, although some cancer cell lines, such as T cell and B cell lymphoma cell lines, have a constitutive expression of PD-L1, this result suggests that the majority of cancer cell lines show a quite low intrinsic PD-L1 expression.

**Figure 3:**
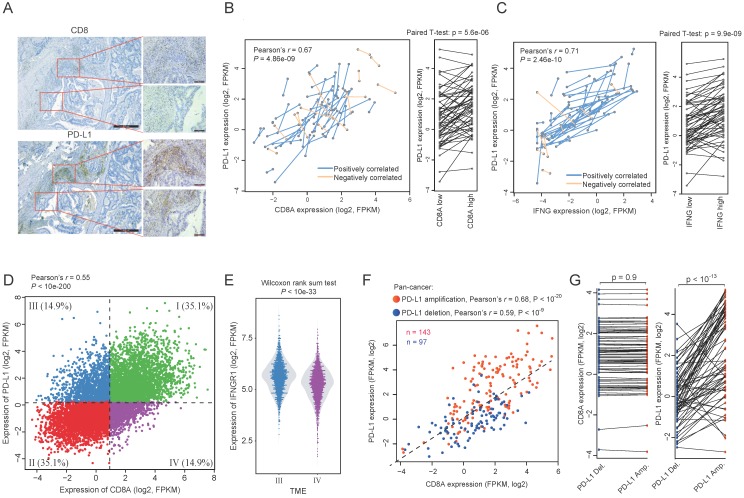
** Regulatory events of the association between PD-L1 expression and CD8A.** (A) A representative IHC staining of colon tumor sample showing the intratumoral correlation between CD8 and PD-L1. (B) Correlation between PD-L1 expression and CD8A expression across tumor samples. Samples from different portions of the same tumor are connected by lines. Blue lines indicate the sample pairs where high CD8A expression is associated with high PD-L1 expression, whereas orange lines indicate the sample pairs where high CD8A expression is associated with low PD-L1 expression. Right panel shows a pairwise comparison of samples from different portions of the same tumor. (C) Same as (B) but shown for the correlation between the expression of PD-L1 and IFN-γ. (D) Scatter plot of the expression of PD-L1 and CD8A across pan-cancer tumors. TME I-IV are shown by different colors. (E) Violin plots of IFNGR1 expression for TME-III tumors and TME-IV tumors. (F) Scatter plot of the expression of PD-L1 and CD8A according to PD-L1 amplification (red) or deletion (blue). (G) Pairwise comparison of the expression of CD8A and PD-L1 between tumors with extremely similar expression of CD8A but different copy number status of PD-L1.

To test this in protein level, we measured the expression of PD-L1 and CD8 using IHC in 61 formalin-fixed paraffin-embedded cancer tissues (Figure 2B). However, we didn't find any tumor samples with constitutively high expression of PD-L1 but low infiltration of CD8+ T cells (Figure 2C). On the contrary, there is a strong correlation between the expression level of PD-L1 and CD8A in those cancer tissues (Pearson's r = 0.64, p < 10^-7^). This further suggests that reactive expression of PD-L1 by CTL is primary in tumors whereas constitute expression of PD-L1 is uncommon. This is also seen in TCGA RNAseq data. We found that the expression of PD-L1 shows a strong correlation with CTL across 9,264 tumors from different cancer types (Pearson's r = -0.50, p < 10^-200^; Figure 3D and Supplementary Figure 3A) and the infiltration with different immune cells (Supplementary Figure 3B).

### Intratumoral reactive expression of PD-L1

In tumor samples, we observed many areas with focal expression of PD-L1 and CD8A (Figure 3A), suggesting that the CTL-reactive expression of PD-L1 also exists at the intratumor level. To explore this in detail, we identified 59 pairs of tumor samples from TCGA pan-cancer resources, with each pair from different portions of the same tumor sample. We observed considerable variations of CD8A expression between different portions of the same tumor sample (Figure 3B). The variation of CD8A expression is significantly correlated with the corresponding PD-L1 expression. Among these 59 pairs of samples, we identified 44 pairs for which higher CD8A expression is associated with higher PD-L1 expression (positively correlated; p < 10-3, binomial test). A pair-wise comparison between the two portions of the same tumor also shows that PD-L1 expression is significantly increased in the portions with higher CD8A expression (Left panel of Figure 3B). Given that the reactive expression of PD-L1 is induced by IFN-γ, we also tested the correlation between the expression of INFG and PD-L1 and found that they were positively correlated in 51 out of the 59 pairs of samples (Figure 3C; p < 10^-8^, binomial test).

### Genetic events regulating the reactive expression of PD-L1 in tumors

To investigate the factors that may regulate the reactive efficiency of PD-L1 expression, we divided tumors into four tumor microenvironment (TME) types according to the median expression of PD-L1 and CD8A, in which TME III is CD8A-low and PD-L1-high whereas TME IV is CD8A-high and PD-L1-low (Figure 3D). We then compared the expression of T cell response related genes between these two types of tumors. Interestingly, although the CTL level is lower in TME III tumors, we observed significantly higher expression of IFNGR-JAK-STAT genes in these tumors compared with tumors in TME IV, including IFNGR1 (Figure 3E), JAK1, JAK2, STAT1 and STAT3 (Wilcoxon rank sum test, p < 0.01 for all the genes). Given the fact that IFN-γ can induce the reactive expression of PD-L1 via the IFNGR-JAK-STAT axis 18, this may explains why TME III has a relatively higher PD-L1 expression.

In addition, we found that TME-III contains more PD-L1 amplified tumors (n = 30) but less PD-L1 deleted tumors (n = 8), whereas TME-IV contains less PD-L1 amplified tumors (n = 2) but more PD-L1 deleted tumors (n = 19) (Fisher exact test, p < 0.001). To test whether PD-L1 amplification results in constitutive expression, we plotted the correlation between the expression of PD-L1 and CD8A in PD-L1 gene amplified tumors (Figure 3F). We observed a stronger correlation between the expression of PD-L1 and CD8A for tumors with PD-L1 amplification than tumors with PD-L1 deletion, which suggests a clearly CTL-reactive expression rather than constitutive expression. To further investigate whether PD-L1 amplification plays an independent role in regulating PD-L1 reactive expression, we ordered the samples by the expression of CD8A, and selected 70 tumor pairs having almost the same CD8A expression but different copy number status of PD-L1 (Figure. 3G). PD-L1 amplified tumors have significantly higher PD-L1 expression than their paired control tumors (pairwise T test, p < 10^-13^). This result indicates that the reactive expression of PD-L1 expression could be regulated by genetic events. However, the constitutive expression of PD-L1, though has been observed in occasional cases 19, is relatively uncommon in tumors.

### The independent prognostic value of PD-L1 in immunotherapy dataset

First, using Cox-PH model, we evaluated the independent prognostic value of PD-L1 adjusted for CD8A expression. We found that most cancers show a higher HR of PD-L1 expression adjusted for CD8A by multivariate Cox-Ph model, with above half of them larger than 1 (Table 1), indicating that PD-L1 are more likely to be associated with poor outcome after adjusting for CD8A expression. For example, in head & neck cancer (HNSC), PD-L1 expression is significantly associated with poor outcome only after adjusting for CD8A expression (HR = 1.20[95%CI: 1.02-1.41], p < 0.03). Interestingly, HNCN is the cancer where PD-L1 is most frequently amplified.

**Figure 4:**
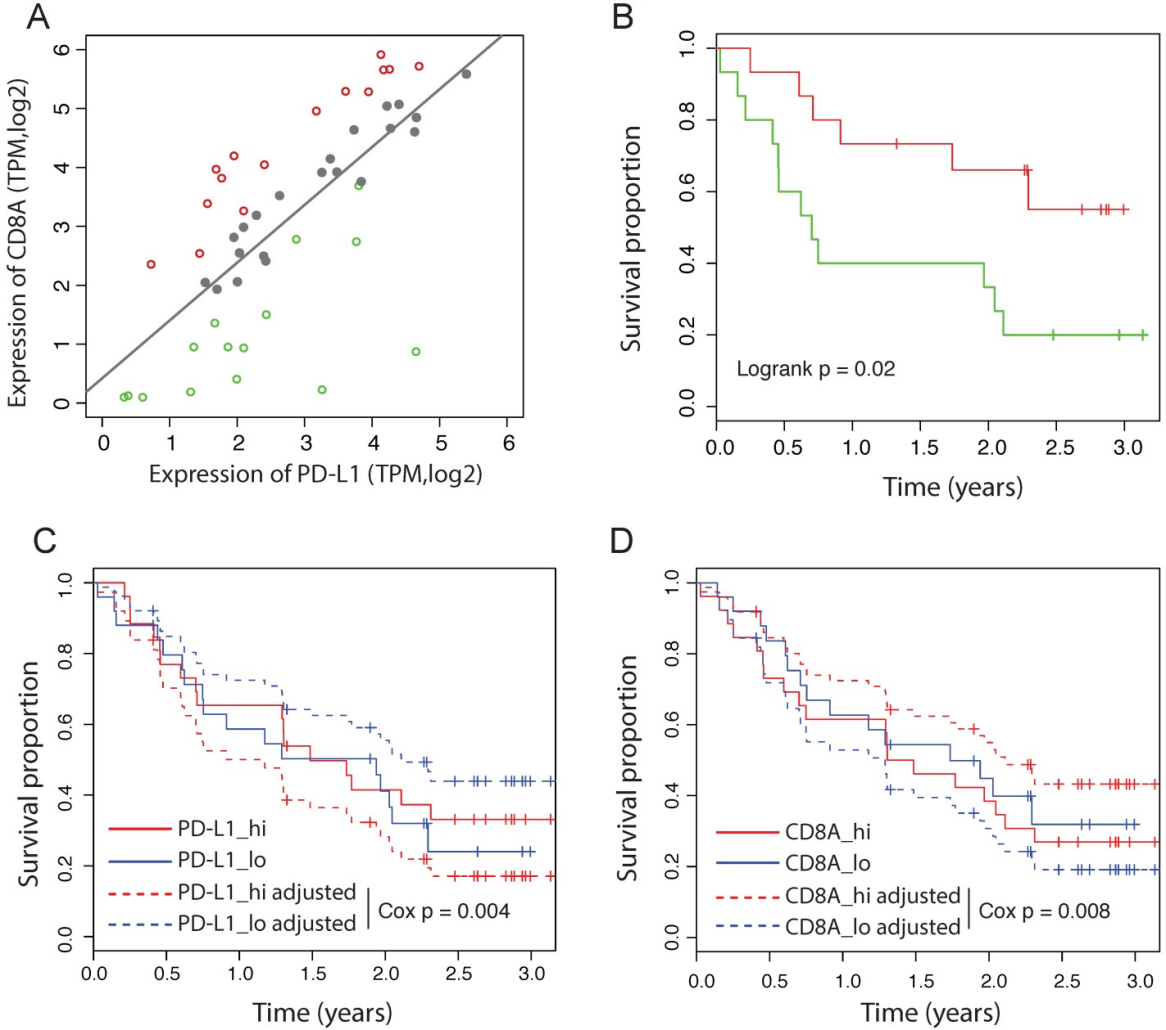
** Independent prognostic values of PD-L1 in immunotherapy dataset.** (A) Correlation between the expression of CD8A and PD-L1 in melanoma patients treated by nivolumab. Dashed line represents linear regression model. Patients are divided into the high efficient reactive group (red), the expected reactive group (grey) and the high efficient reactive group (green). (B) Survival curves of melanoma patients between the low and high efficient reactive group. (C) Survival curves of PD-L1 before and after adjusted for CD8A expession in melanoma received nivolumab. Adjusted survival curves are generated by multivariate Cox_PH model. Patients are divided by the median value. (D) Survival curves of CD8A before and after adjusted for PD-L1 expression in melanoma received nivolumab.

Some of the recent immunotherapy studies failed to observe a significant association between PD-L1 expression and the response to immunotherapy, including the PD-L1 blockade therapy 20-23. We hypothesize that the reactive efficiency of PD-L1 expression might be more important than the absolute PD-L1 expression level to predict the response to PD-1/PD-L1 blockade. To test this idea, we analyzed the RNAseq data from 51 patients with primary advanced melanoma treated with nivolumab 23. Notably, in this dataset, neither the expression of PD-L1 nor CD8A is significantly associated with the outcome by the univariate Cox-PH model (PD-L1: HR = 0.98 [95%CI: 0.62-1.53], Cox p = 0.93; CD8A: HR = 1.05 [95%CI: 0.77-1.44], Cox p = 0.74). We fitted the expression of CD8A and PD-L1 using a linear model, and divided patients into three groups according to their distance to the predicted value of the linear model (Figure 4A). Tumors on the left of the line have a less efficient reactive expression of PD-L1 than expected, whereas tumors on the right of the line have a more efficient reactive expression of PD-L1. We found that patients with different reactive efficiency of PD-L1 expression have significantly different outcome (Figure 4B), in contrast to the expression level of PD-L1 that has no significant prognostic value (Figure 4C). Using multivariate Cox-PH analysis, we found that CD8A adjusted for PD-L1 expression is significantly associated with favorable outcome (HR = 0.62 [95%CI: 0.45-0.86], Cox p = 0.004). Consistently, PD-L1 adjusted for CD8A expression is significantly associated with poor outcome (HR = 1.93 [95%CI: 1.18-3.14], Cox p = 0.008). The adjusted survival curves predicted by multivariate Cox-PH model are shown for PD-L1 and CD8A respectively (Figure 4C and 4D), suggesting that the effect of PD-L1 on response to immunotherapy depends more on its reactive efficiency to CTL level than its absolute expression level.

## Discussion

This study demonstrates a simple but important fact that the expression of PD-L1 is primarily reactive rather than constitutive in most tumors. The reactive effect of PD-L1 expression to CTL is much stronger than previously appreciated. It suggests that PD-L1 expression, when being evaluated independently, may have a similar prognostic value with CTL. We have no intention to argue against the numerous previous studies that suggest a poor prognostic value of PD-L1, because prognostic value varies across different cohorts, as we also found for ovarian cancer (Fig. 1B). In addition, it depends on how many patients show a constitutive expression of PD-L1 in the cohort. However, we do hope our study will encourage people to publish unexpected results about the prognostic value of PD-L1 in the future.

Many studies have suggested the association between the aberrant PD-L1 expression and oncogene-driven mechanisms, such as the activation of PI3K-AKT pathway, the amplification of PD-L1 gene and the mutations in BRAF, NAS and PTEN 24-26. Although tumors with these oncogene events may show overexpression of PD-L1, we failed to observe the constitutive expression of PD-L1 in the cancer cell lines. It suggests that PD-L1 expression in these tumors is still reactive, though more efficiently 27. For example, we showed that the amplification of PD-L1 gene only enhances the response to CTL, rather than results in a constitutive expression of PD-L1. It is also possible that tumors with specific oncogenic events have increased CTL, and consequently, the enhanced expression of PD-L1. It has been shown that BRAF inhibition is associated with enhanced antigen expression and high density of TILs 26. A recent study further showed that, when focused on the broad expression of PD-L1 in melanocytes including the cells not surrounded by TILs in the tumor section, there was no correlation between BRAF mutation status and PD-L1 expression 28. Furthermore, PD-L1 was found to be focally expressed by both TILs and melanocytes close to the tumor-host interface 29, indicating a reactive expression of PD-L1 in these tumors.

Therefore, for majority of tumors, our study supports a model of “adaptive immune resistance” 30, in which CTL induces PD-L1 expression that in turn inhibits T cell functions. In this model, PD-L1 expression, as the consequence of CTL instead of the innate capability of tumor cells, plays a crucial role in balancing the tumor microenvironment and would be important in determining the responsiveness of immunotherapy that blocks PD-1/PD-L1 axis. Since the blockade of PD-1/PD-L1 axis tips the balance toward the anti-tumor immunity, tumors that trigger the immune resistance less efficiently are supposed to have a better response. Given the importance of pre-existing anti-tumor immunity in the setting of checkpoint blockade 31, models adjusting for PD-L1 reactive expression might have important clinical implications for anti-PD-1/PD-L1 therapies.

## Supplementary Material

Supplementary figures and tables.

## Figures and Tables

**Table 1:** Prognostic value of PD-L1 expression before and after adjustment of CTL.

Cancer types	Before adjustment		After adjustment	
	HR[95%CI]	p value	HR[95%CI]	p value
BLCA	0.90[0.78-1.04]	0.151	0.97[0.80-1.18]	0.767
BRCA	0.87[0.74-1.02]	0.085	1.01[0.82-1.25]	0.943
CESC	0.87[0.70-1.10]	0.241	1.01[0.80-1.28]	0.913
CRC	0.90[0.75-1.08]	0.263	0.90[0.71-1.15]	0.417
GBM	1.15[0.97-1.37]	0.100	1.14[0.96-1.36]	0.133
HNSC	1.01[0.88-1.16]	0.876	1.20[1.02-1.41]	0.029
KIRC	0.87[0.74-1.03]	0.099	0.82[0.69-0.97]	0.024
LIHC	0.91[0.76-1.10]	0.351	1.03[0.83-1.28]	0.771
LUAD	1.00[0.87-1.14]	0.948	1.07[0.91-1.26]	0.425
LUSC	0.99[0.87-1.14]	0.937	1.01[0.87-1.17]	0.921
OV	0.84[0.74-0.96]	0.008	0.83[0.71-0.97]	0.022
SKCM	0.69[0.60-0.78]	1.942E-08	0.76[0.62-0.93]	0.008
STAD	0.94[0.80-1.10]	0.440	0.89[0.73-1.09]	0.267
THCA	0.83[0.51-1.11]	0.460	0.96[0.57-1.64]	0.889
UCEC	0.90[0.73-1.11]	0.332	1.10[0.85-1.43]	0.452

## Abbreviations

PD-L1: programmed death-ligand 1
CTL: cytotoxic T lymphocyte
TILs: tumor-infiltrating leucocytes
NSCLC: non-small cell lung cancer
CRC: colorectal cancer
MSI: microsatellite instable
