# Supplementary material for: Reactive versus Constitutive: Reconcile the Controversial Results about the Prognostic Value of PD-L1 Expression in cancer
Source: Int J Biol Sci. 2019 Jul 21;15(9):1933–41. doi: 10.7150/ijbs.33297 (PMC6743303; doi:10.7150/ijbs.33297)
Supplement: Supplementary file 1 — Supplementary figures and tables. [file ijbsv15p1933s1.pdf]

Supplementary table 1. The clinical information of the patients

The types of cancer	age			TNM stage				neo-adjuvant chemotherapies	
	n=61	<50	≥50	I	II	III	IV	Yes	No
colorectal cancer	41	12	29	4	20	16	1	0	41
breast cancer	16	7	9	2	8	6	0	0	16
lung cancer	3	1	2	0	1	2	0	0	3
esophageal cancer	1	1	0	0	0	1	0	0	1

Supplementary Table 2. The sample barcode of TCGA tumor samples.

Tumor sample ID	Portion A	Portion B
1	TCGA.44.2656.01A.02R.0946.07	TCGA.44.2656.01B.06R.A277.07
1	TCGA.44.2656.01A.02R.A278.07	TCGA.44.2656.01B.06R.A277.07
2	TCGA.44.2662.01A.01R.0946.07	TCGA.44.2662.01B.02R.A277.07
2	TCGA.44.2662.01A.01R.A278.07	TCGA.44.2662.01B.02R.A277.07
3	TCGA.44.2665.01A.01R.0946.07	TCGA.44.2665.01B.06R.A277.07
3	TCGA.44.2665.01A.01R.A278.07	TCGA.44.2665.01B.06R.A277.07
4	TCGA.44.2666.01A.01R.0946.07	TCGA.44.2666.01B.02R.A277.07
4	TCGA.44.2666.01A.01R.A278.07	TCGA.44.2666.01B.02R.A277.07
5	TCGA.44.2668.01A.01R.0946.07	TCGA.44.2668.01B.02R.A277.07
5	TCGA.44.2668.01A.01R.A278.07	TCGA.44.2668.01B.02R.A277.07
6	TCGA.44.3918.01A.01R.1107.07	TCGA.44.3918.01B.02R.A277.07
6	TCGA.44.3918.01A.01R.A278.07	TCGA.44.3918.01B.02R.A277.07
7	TCGA.44.4112.01A.01R.1107.07	TCGA.44.4112.01B.06R.A277.07
7	TCGA.44.4112.01A.01R.A278.07	TCGA.44.4112.01B.06R.A277.07
8	TCGA.44.5645.01A.01R.1628.07	TCGA.44.5645.01B.04R.A277.07
8	TCGA.44.5645.01A.01R.A278.07	TCGA.44.5645.01B.04R.A277.07
9	TCGA.44.6146.01A.11R.1755.07	TCGA.44.6146.01B.04R.A277.07
9	TCGA.44.6146.01A.11R.A278.07	TCGA.44.6146.01B.04R.A277.07
10	TCGA.44.6147.01A.11R.1755.07	TCGA.44.6147.01B.06R.A277.07
10	TCGA.44.6147.01A.11R.A278.07	TCGA.44.6147.01B.06R.A277.07
11	TCGA.44.6775.01A.11R.1858.07	TCGA.44.6775.01C.02R.A277.07
11	TCGA.44.6775.01A.11R.A278.07	TCGA.44.6775.01C.02R.A277.07
12	TCGA.A6.2674.01A.02R.0821.07	TCGA.A6.2674.01B.04R.A277.07
12	TCGA.A6.2674.01A.02R.A278.07	TCGA.A6.2674.01B.04R.A277.07
13	TCGA.A6.2677.01A.01R.0821.07	TCGA.A6.2677.01B.02R.A277.07
13	TCGA.A6.2677.01A.01R.A278.07	TCGA.A6.2677.01B.02R.A277.07

14	TCGA.A6.2684.01A.01R.1410.07	TCGA.A6.2684.01C.08R.A277.07
14	TCGA.A6.2684.01A.01R.A278.07	TCGA.A6.2684.01C.08R.A277.07
15	TCGA.A6.3809.01A.01R.1022.07	TCGA.A6.3809.01B.04R.A277.07
15	TCGA.A6.3809.01A.01R.A278.07	TCGA.A6.3809.01B.04R.A277.07
16	TCGA.A6.3810.01A.01R.1022.07	TCGA.A6.3810.01B.04R.A277.07
16	TCGA.A6.3810.01A.01R.A278.07	TCGA.A6.3810.01B.04R.A277.07
17	TCGA.A6.5656.01A.21R.1839.07	TCGA.A6.5656.01B.02R.A277.07
17	TCGA.A6.5656.01A.21R.A278.07	TCGA.A6.5656.01B.02R.A277.07
18	TCGA.A6.5659.01A.01R.1653.07	TCGA.A6.5659.01B.04R.A277.07
18	TCGA.A6.5659.01A.01R.A278.07	TCGA.A6.5659.01B.04R.A277.07
19	TCGA.A6.6650.01A.11R.1774.07	TCGA.A6.6650.01B.02R.A277.07
19	TCGA.A6.6650.01A.11R.A278.07	TCGA.A6.6650.01B.02R.A277.07
20	TCGA.A6.6780.01A.11R.1839.07	TCGA.A6.6780.01B.04R.A277.07
20	TCGA.A6.6780.01A.11R.A278.07	TCGA.A6.6780.01B.04R.A277.07
21	TCGA.A6.6781.01A.22R.1928.07	TCGA.A6.6781.01B.06R.A277.07
21	TCGA.A6.6781.01A.22R.A278.07	TCGA.A6.6781.01B.06R.A277.07
22	TCGA.A7.A0DB.01A.11R.A00Z.07	TCGA.A7.A0DB.01C.02R.A277.07
22	TCGA.A7.A0DB.01A.11R.A277.07	TCGA.A7.A0DB.01C.02R.A277.07
23	TCGA.A7.A13D.01A.13R.A12P.07	TCGA.A7.A13D.01B.04R.A277.07
23	TCGA.A7.A13D.01A.13R.A277.07	TCGA.A7.A13D.01B.04R.A277.07
24	TCGA.A7.A13E.01A.11R.A12P.07	TCGA.A7.A13E.01B.06R.A277.07
24	TCGA.A7.A13E.01A.11R.A277.07	TCGA.A7.A13E.01B.06R.A277.07
25	TCGA.A7.A26E.01A.11R.A169.07	TCGA.A7.A26E.01B.06R.A277.07
25	TCGA.A7.A26E.01A.11R.A277.07	TCGA.A7.A26E.01B.06R.A277.07
26	TCGA.A7.A26J.01A.11R.A169.07	TCGA.A7.A26J.01B.02R.A277.07
26	TCGA.A7.A26J.01A.11R.A277.07	TCGA.A7.A26J.01B.02R.A277.07
27	TCGA.B2.3923.01A.02R.1325.07	TCGA.B2.3923.01B.10R.A277.07
27	TCGA.B2.3923.01A.02R.A277.07	TCGA.B2.3923.01B.10R.A277.07
28	TCGA.B2.3924.01A.02R.1325.07	TCGA.B2.3924.01B.03R.A277.07
28	TCGA.B2.3924.01A.02R.A277.07	TCGA.B2.3924.01B.03R.A277.07
29	TCGA.B2.5633.01A.01R.1541.07	TCGA.B2.5633.01B.04R.A277.07
29	TCGA.B2.5633.01A.01R.A277.07	TCGA.B2.5633.01B.04R.A277.07
30	TCGA.B2.5635.01A.01R.1541.07	TCGA.B2.5635.01B.04R.A277.07
30	TCGA.B2.5635.01A.01R.A277.07	TCGA.B2.5635.01B.04R.A277.07
31	TCGA.BK.A0CA.01A.21R.A118.07	TCGA.BK.A0CA.01B.02R.A277.07
31	TCGA.BK.A0CA.01A.21R.A277.07	TCGA.BK.A0CA.01B.02R.A277.07
32	TCGA.BK.A0CC.01A.21R.A16W.07	TCGA.BK.A0CC.01B.04R.A277.07
32	TCGA.BK.A0CC.01A.21R.A277.07	TCGA.BK.A0CC.01B.04R.A277.07
33	TCGA.BK.A139.01A.11R.A118.07	TCGA.BK.A139.01C.08R.A277.07
33	TCGA.BK.A139.01A.11R.A277.07	TCGA.BK.A139.01C.08R.A277.07
34	TCGA.BK.A26L.01A.11R.A16F.07	TCGA.BK.A26L.01C.04R.A277.07
34	TCGA.BK.A26L.01A.11R.A277.07	TCGA.BK.A26L.01C.04R.A277.07
35	TCGA.BL.A0C8.01A.11R.A10U.07	TCGA.BL.A0C8.01B.04R.A277.07
35	TCGA.BL.A0C8.01A.11R.A277.07	TCGA.BL.A0C8.01B.04R.A277.07

36	TCGA.BL.A13I.01A.11R.A13Y.07	TCGA.BL.A13I.01B.04R.A277.07
36	TCGA.BL.A13I.01A.11R.A277.07	TCGA.BL.A13I.01B.04R.A277.07
37	TCGA.BL.A13J.01A.11R.A10U.07	TCGA.BL.A13J.01B.04R.A277.07
37	TCGA.BL.A13J.01A.11R.A277.07	TCGA.BL.A13J.01B.04R.A277.07
38	TCGA.06.0211.01A.01R.1849.01	TCGA.06.0211.01B.01R.1849.01
39	TCGA.23.1023.01A.02R.1564.13	TCGA.23.1023.01R.01R.1564.13
40	TCGA.44.3917.01A.01R.A278.07	TCGA.44.3917.01B.02R.A277.07
41	TCGA.A6.2672.01A.01R.0826.07	TCGA.A6.2672.01B.03R.2302.07
42	TCGA.A6.5661.01A.01R.1653.07	TCGA.A6.5661.01B.05R.2302.07
43	TCGA.A6.5665.01A.01R.1653.07	TCGA.A6.5665.01B.03R.2302.07
44	TCGA.A7.A0DC.01A.11R.A00Z.07	TCGA.A7.A0DC.01B.04R.A22O.07
45	TCGA.A7.A13G.01A.11R.A13Q.07	TCGA.A7.A13G.01B.04R.A22O.07
46	TCGA.A7.A26F.01A.21R.A169.07	TCGA.A7.A26F.01B.04R.A22O.07
47	TCGA.A7.A26I.01A.11R.A169.07	TCGA.A7.A26I.01B.06R.A22O.07
48	TCGA.AC.A2QH.01A.11R.A18M.07	TCGA.AC.A2QH.01B.04R.A22O.07
49	TCGA.AC.A3OD.01A.11R.A21T.07	TCGA.AC.A3OD.01B.06R.A22O.07
50	TCGA.AC.A3QQ.01A.11R.A22K.07	TCGA.AC.A3QQ.01B.06R.A22O.07
51	TCGA.DD.AACA.02A.11R.A41C.07	TCGA.DD.AACA.02B.11R.A41C.07
52	TCGA.DU.6404.02A.21R.A36H.07	TCGA.DU.6404.02B.11R.A36H.07
53	TCGA.DU.6407.02A.12R.A36H.07	TCGA.DU.6407.02B.11R.A36H.07
54	TCGA.FG.5965.02A.11R.A29R.07	TCGA.FG.5965.02B.11R.A29R.07
55	TCGA.HC.7740.01A.11R.2118.07	TCGA.HC.7740.01B.04R.2302.07
56	TCGA.HC.8258.01A.11R.2263.07	TCGA.HC.8258.01B.05R.2302.07
57	TCGA.HC.8261.01A.11R.2263.07	TCGA.HC.8261.01B.05R.2302.07
58	TCGA.HC.8265.01A.11R.2263.07	TCGA.HC.8265.01B.04R.2302.07
59	TCGA.TQ.A7RK.02A.11R.A36H.07	TCGA.TQ.A7RK.02B.11R.A40A.07

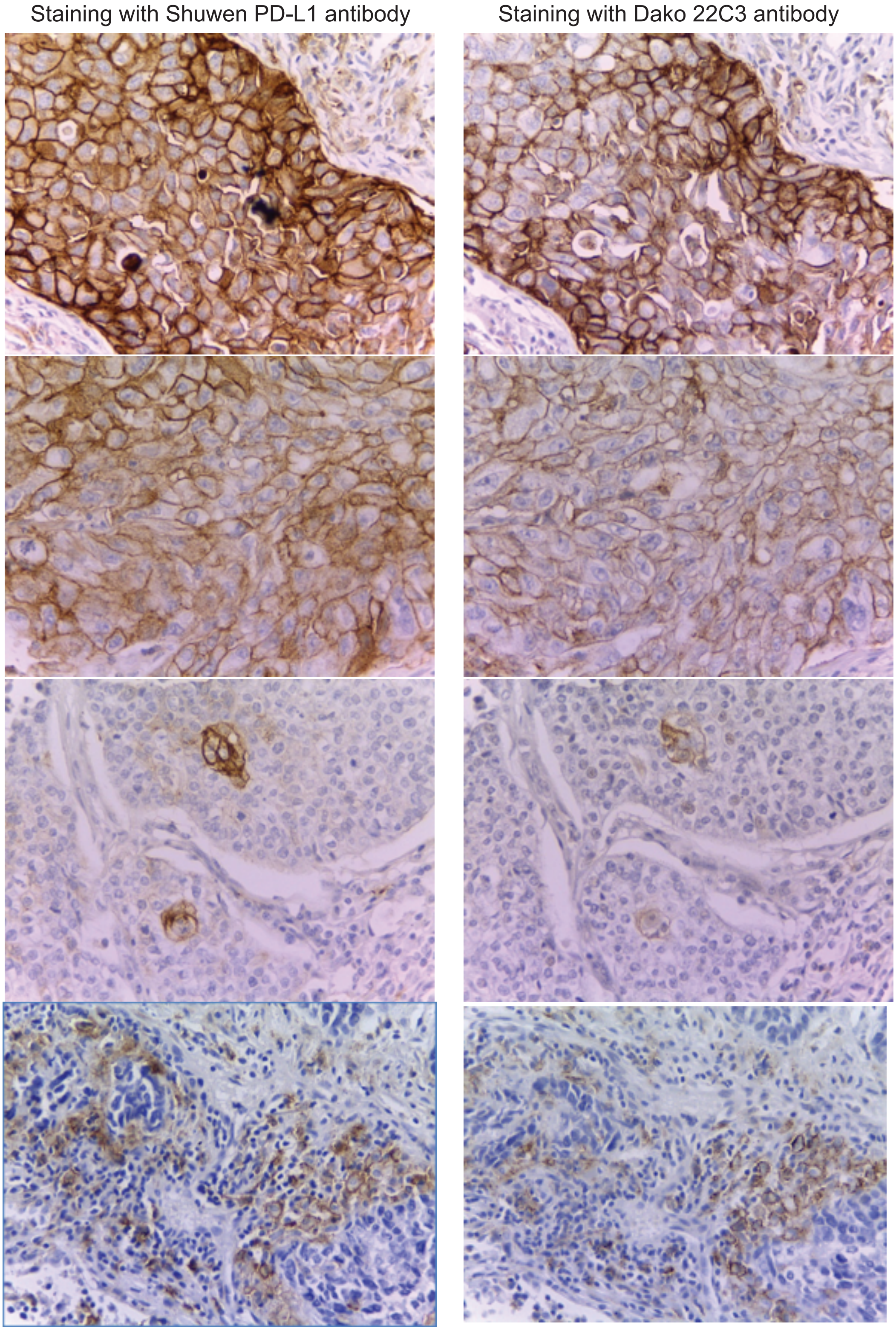

Figure S1 | Validation of PD-L1 antibody (Shuwen Biotech Company) with Dako 22C3 antibody.

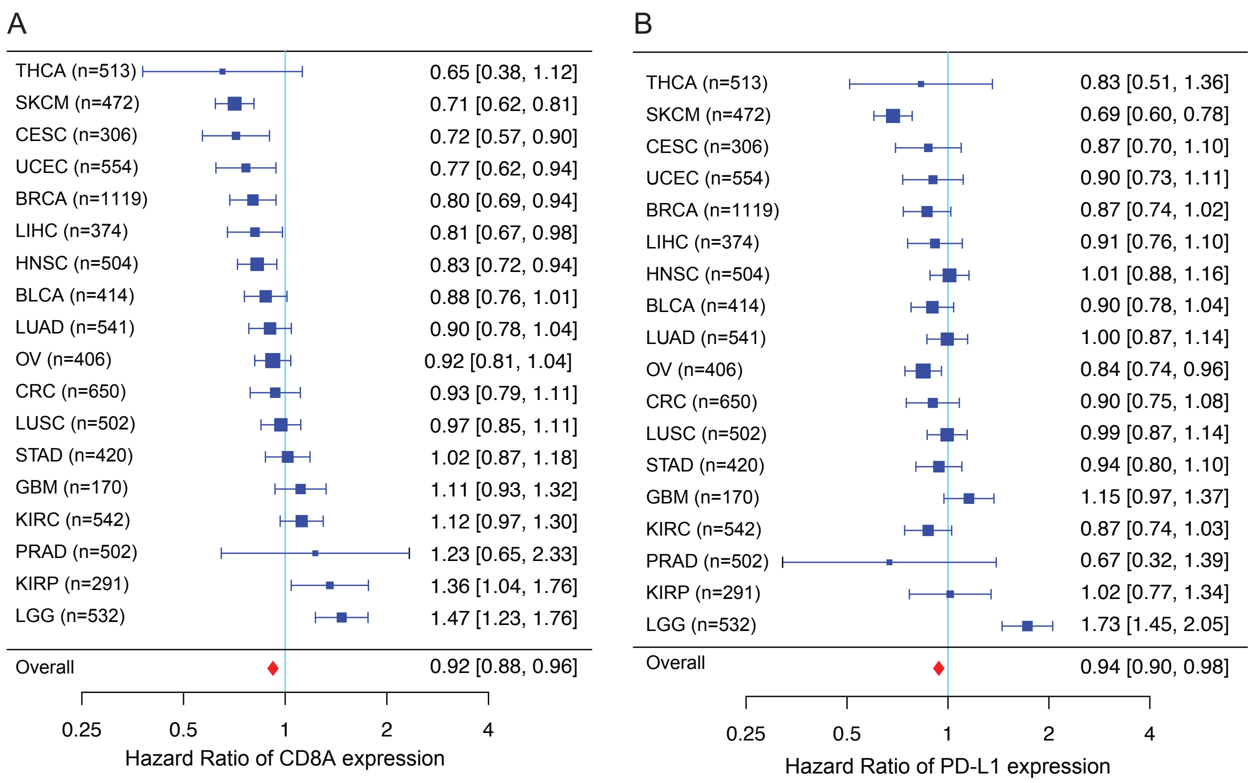

Figure S2 | Prognostic evaluation of PD-L1 and CD8A. (**A**) Forest plot visualizing the hazard ratios (HRs) of univariate Cox proportional regression analyses of CD8A expression in 18 solid cancer types. The red diamond shows the fixed-effects meta-analysis summary of HRs over 18 cancer types. (**B**) Same as (**A**) but evaluated for PD-L1 expression.

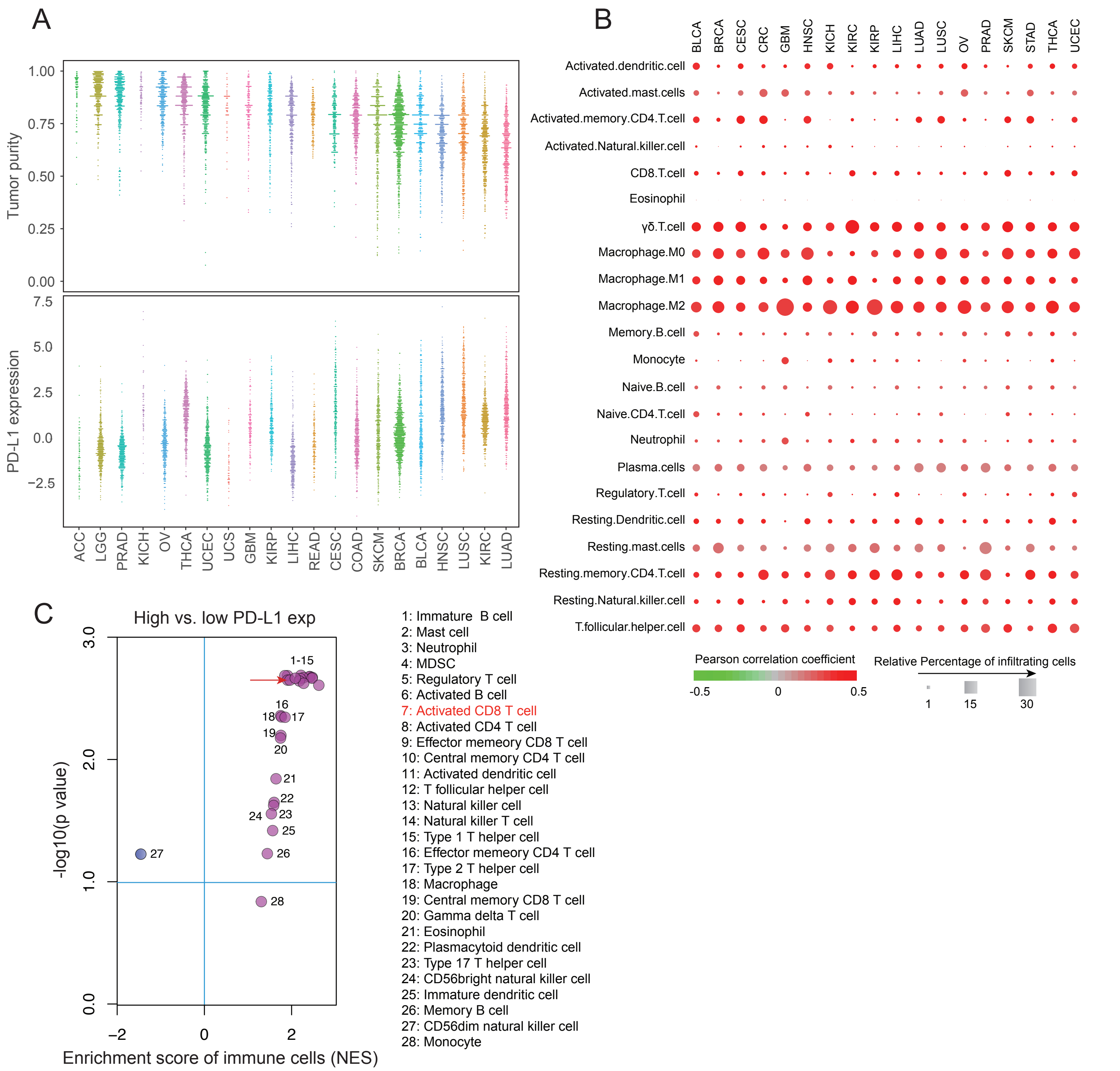

Figure S3 | Pan-cancer association between PD-L1 expression and infiltrating immune cells. (A) Violin plots of tumor purity (top panel) and PD-L1 expression (bottom panel) in 21 cancer types. (B) Bubble plot of the coexpression between PD-L1 and marker genes of 22 immune subpopulations across 18 solid cancers (sorted alphabetically). The size of the circles gives the average value of relative percentage of immune subpopulations within each cancer type, and the color indicates the average Pearson correlation coefficient between the expression of PD-L1 and marker genes of each immune subpopulation. The strongest correlation is found with CD8 T cell. (C) GSEA of immune cells in tumors with high versus low expression of PD-L1. Volcano plots for the enrichment (purple) or depletion (blue) of each immune cell types in pan-cancer solid tumor samples with high versus low PD-L1 expression. The enrichment score is calculated based on the averaged normalized enrichment score (NES) of GSEA.
